# Oro-ruminal sampling device and technique for rapid collection of rumen content and improved recovery of solid fractions for microbiome analysis

**DOI:** 10.3168/jdsc.2023-0536

**Published:** 2024-05-31

**Authors:** F.E. Miccoli, R.I. Galarza, N. Juliano, S. Ferreyra, S. Maresca, S. López-Valiente, L.D. Guerrero, R.A. Palladino, R.I. Albornoz

**Affiliations:** 1 Facultad de Ciencias Agrarias, Instituto de Investigacion en Produccion Agropecuaria, Ambiente y Salud (IIPAAS), Universidad Nacional de Lomas de Zamora (UNLZ), Llavallol, 1836, Buenos Aires, Argentina; 2 Departamento de Producción Animal, Universidad de Buenos Aires (UBA), Caba, C1417DSE, Argentina; 3 Estacion Experimental Agropecuaria del Instituto Nacional de Tecnología Agropecuaria, EEA Cuenca del Salado–INTA, Rauch 7203, Buenos Aires, Argentina; 4 Consejo Nacional de Investigaciones Científicas y Técnicas (CONICET), Caba, C1417DSE, Argentina; 5 Instituto de Investigaciones en Ingeniería Genética y Biología Molecular “Dr. Héctor N. Torres” (INGEBI), CABA, C1428ADN, Buenos Aires, Argentina; 6 Dairy Australia, Melbourne, VIC 3006, Australia

## Abstract

Rumen fistulation is a widely used procedure that allows for collection of ruminal contents. However, fistulation is an invasive and costly procedure that generally limits the number of animals that can be recruited for experiments, thus encouraging the use of alternative techniques such as the intra-esophageal tube technique. One of the challenges of this technique is the limited ability to collect solid fractions from the rumen content pool, which may affect the microbial community structure in the sample, particularly affecting the recovery and characterization of solid adherent bacteria. We developed an intra-esophageal tube rumen sampling technique and device referred to as the “Rumen Sampler MG” with the aim of increasing the recovery of solid fractions from rumen content compared with other oro-ruminal sampling methods. The Rumen Sampler MG device consists of a manual pump fitted with a barometer and an intra-esophageal flexible polyvinyl chloride (PVC) tube with beveled terminal edge allowing for minimal clogging of the sampling tubing and a rapid flux of contents being sampled. Nine lactating Holstein-Friesian dairy cows (554.6 ± 25.2 kg of BW; 8.3 ± 3.3 DIM; ± SD) were recruited to evaluate the proposed method. During the procedure, animals were safely restrained in a chute and administered with a low dosage of a neuroleptic drug to reduce animal stress during sampling. An endoscopic camera was inserted into the reticulo-rumen through the esophagus to identify the sampling location and determine the length of the sampling tube necessary to reach the desired location. Following, the intra-esophageal sampling tube connected to a manual pump was inserted for collection of rumen contents. Samples collected did not present visual evidence of saliva contamination (e.g., high viscosity) and their pH ranged within expected values (6.33–7.04) for samples collected from the reticulo-rumen. Each sample contained 35% to 40% wet solids volume. Individual dry matter intake and milk production of cows continued to increase after sampling as expected for cows in the early postpartum period, suggesting that the sampling procedure did not affect cow performance. Results from microbiome analysis of rumen content samples suggest that the relative abundances of the main bacterial phyla are consistent with those from samples collected from dairy cows via rumen fistula in previous studies. The device and technique proposed allow for adequate samples of ruminal liquid and solid contents to be collected for microbiome analysis without disruption of animal performance.

## Highlights

• The proposed rumen content sampling device and technique increases the recovery of solids.
• Samples collected using this method are suitable for rumen microbiome analysis.
• The device incorporates a manual pump that allows for the sampling method to be implemented without electricity.

## Graphical Abstract

**Figure:**
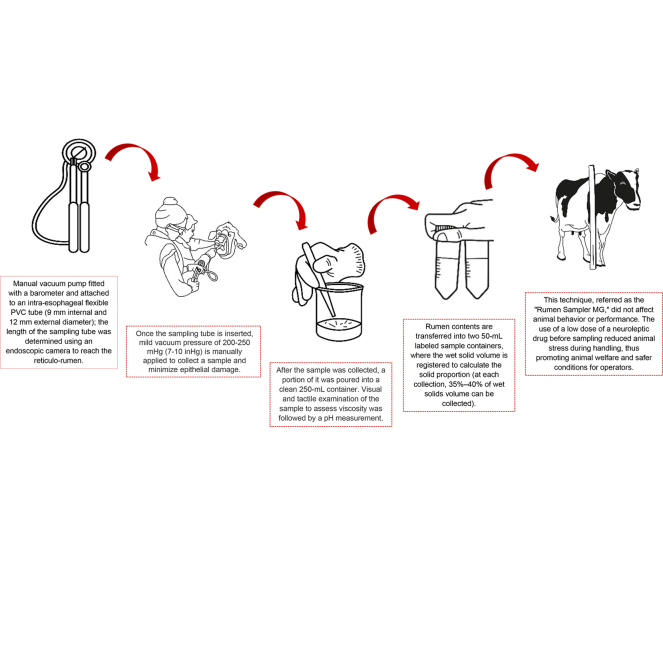
**Summary:** Intra-esophageal tube techniques for rumen content sampling can limit the collection of solids from the rumen content pool, which may affect the recovery and characterization of solid adherent bacteria. We developed an intra-esophageal tube rumen sampling technique and device to increase the recovery of solid fractions compared with other oro-ruminal sampling methods. The relative abundances of the main bacterial phyla found in samples collected using our device and technique are consistent with those from samples previously collected from dairy cows via rumen fistula. The proposed intra-esophageal tube technique for improved microbiome analysis did not affect animal performance, suggesting that the technique imposed minimal stress on the animal.

Rumen microbiome research requires rumen sampling techniques that allow for adequate characterization of microbial communities and interactions among themselves, and with the host and diet. Ruminal fistulation dates back to the first in vivo study conducted for the evaluation of rumen pH (Smith, 1941) and represents one of the main techniques for collection of ruminal content samples. Since then, this technique has also been used for characterization of feeds and diets, nutrient degradation kinetics, gas production kinetics, and metagenomic analysis (Klopp et al., 2018). Morgavi et al. (2013) refers to ruminants as “the super ruminant organism” to characterize the complexity of the pool of microbial genes and enzymes residing in the rumen. Despite concerns about ruminal fistulation affecting rumen physiology and function, previous studies have shown no detrimental effects on animal feed intake or daily weight gain (Moate et al., 2008; Kristensen et al., 2010). However, rumen fistulation is an invasive, costly, and time-consuming technique, typically limiting the number of animals used for the study of the rumen microbiome, thus encouraging the use and development of alternative methodologies.

The oro-ruminal or intra-esophageal tube techniques have been successfully used in ruminants for collection of ruminal content samples (Geishauser, 1993; Moate et al., 2014). However, Klopp et al. (2018) have identified challenges with oro-ruminal techniques, such as potential for saliva contamination in samples, limited control over sampling location, and obstruction of the sampling tube, which can limit collection of representative solid fractions from the site of extraction. Decreased collection of rumen solids may influence the microbial community structure in the sample given the specificity of solid attached or associated communities such as *Fibrobacter*, *Prevotella*, *Pseudobutyrivibrio*, *Ruminococcus*, and *BF311* (Klevenhusen et al., 2017). Studies with dairy cows (Henderson et al., 2013; Paz et al., 2016) and small ruminants (Ramos-Morales et al., 2014) reported similar microbiome composition between rumen content samples collected via cannula and an intra-esophageal sampling technique, except for abundance of some groups within *Clostridiales* and *Methanobrevibacter* (Henderson et al., 2013), and some fibrolytic genera (Ramos-Morales et al., 2014).

We developed a modified oro-ruminal sampling device and technique from previously reported methods (Henderson et al., 2013; Ramos-Morales et al., 2014; Paz et al., 2016; Klopp et al., 2018) referred to as the “Rumen Sampler MG” with the aim of increasing the recovery of rumen content solid fractions. In comparison with other oro-ruminal sampling methods, the proposed method increases the recovery of rumen solid fractions, improves the accuracy of sampling location, and promotes safer conditions for the animal and operators. In addition, the proposed device contains a manual pump, which allows for this method to be implemented in areas or facilities without access to electric power; however, the pump only allows a volume of ∼100 mL of rumen content to be collected each time the procedure is applied, making it unsuitable if larger volumes need to be extracted from each animal.

The Rumen Sampler MG consists of a manual pump (professional hand-held vacuum pump, Eurotech, Germany) fitted with a barometer and a short flexible polyvinyl chloride (**PVC**) tube (6 mm internal and 9 mm external diameter × 60 mm length) attached to an intra-esophageal flexible sampling PVC tube (9 mm internal and 12 mm external diameter; Figure 1). The short PVC tube is used as an adapter between the extreme end of the pump and the PVC sampling tube, allowing for easy attachment or detachment of the sampling tube as needed. The length of the sampling tube can vary according to the animal characteristics, and this is described in more detail further below.

**Figure 1:**
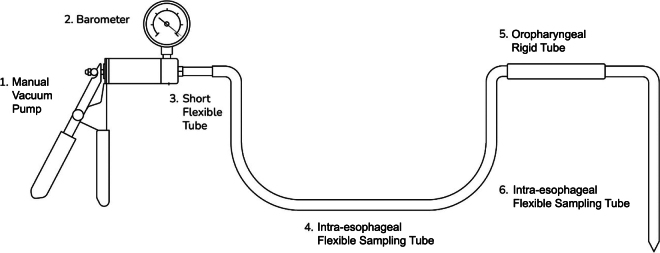
Intra-esophageal tube rumen sampling device used in the technique referred to as “Rumen Sampler MG” for improved recovery of solid fractions for microbiome analysis.

Before sampling, animals are restrained in a chute, and a low dosage of a neuroleptic drug is applied to mitigate some of the inherent stress associated with animal handling and the procedure to increase safety for the animal and operators. Once the animal is restrained and calm, a rigid PVC tube (4 cm × 20 cm) is inserted over the tongue for easy insertion of the endoscopic camera and sampling tubing into the esophagus and to prevent damage from chewing. An endoscopic camera (Trinidad Wolf 2.5 mm Endoscope Camera Mini USB–Android Endoscope, China) fitted within a flexible PVC tube is inserted through the rigid tube and into the reticulo-rumen through the esophagus to identify the sampling location and determine the length of the sampling tube required to reach the site of extraction. If there is uniformity in frame size and breed between animals being sampled, the camera should only be used on the first few animals as the distance from the mouth to the sampling location should be similar. Once the sampling location is reached, the camera is removed and the length to the targeted location is recorded, with an additional length of 30 cm added to the sampling tube to allow for handling of the manual pump. The oro-ruminal tube has a beveled terminal edge without orifices on the tube terminal side walls and it is not connected to any suction strainer as some of the previous devices reported in the literature (Ramos-Morales et al., 2014; Paz et al., 2016). This allows for increased collection of solid fractions and a rapid flux of contents and minimal clogging at each extraction.

An experiment was conducted to evaluate the proposed device and procedure and its effects on indirect indicators of animal welfare (e.g., milk yield and feed intake) at the Agriculture Faculty of Universidad Nacional de Lomas de Zamora Experimental Dairy Farm, with all procedures approved by the Animal Welfare Committee of National University of Lomas de Zamora, Buenos Aires, Argentina (RES CAA 123/17–FCA UNLZ).

Nine multiparous lactating Holstein-Friesian dairy cows (554.6 ± 25.2 kg of BW and 8.3 ± 3.3 DIM; ± SD) were enrolled in this experiment and offered a diet with a forage-to-concentrate ratio (DM basis) of 46:54, with the basal forage being corn silage, and the concentrate composed of 44.92% soy hulls, 20.25% corn grain, 30.45% soybean meal, 1.35% urea, 1.125% calcium carbonate, 0.56% MgSO_4_, 1.12% NaCl, and 0.225% mineral-vitamin mix on a DM basis. Diets were offered ad libitum once a day at 0900 h and the technique was performed 5 h after feeding. Cows were restrained in a chute and administered with an intravenous dose of a neuroleptic drug at 0.25 mL per 100 kg of BW (acepromazine 10 mg/mL; ACEDAN, Holliday Scott S. A., Buenos Aires, Argentina) 3 to 5 min before sampling to minimize animal stress and increase safety of the procedure. Subsequently, the animal's head was held in a forward-facing position and a rigid PVC tube (4 × 20 cm) was inserted over the tongue to protect the endoscopic camera and later the flexible intra-esophageal sampling tube from chewing, also allowing for an easy insertion and removal of both the camera and sampling tube. The endoscopic camera was only used on the first 3 animals to visually identify once the tube reached the reticulo-rumen and determine the length of the intra-esophageal tube required for sampling. The length of the tube required for sampling in the current study was 270 cm. Once the sampling tube was inserted, mild vacuum pressure of 200 to 250 mHg (7 to 10 inHg) was carefully applied manually to minimize potential risk of epithelial damage and sustained to collect the sample within the tubing. After the sample was collected, a portion was poured into a clean 250-mL beaker for an inspection of saliva contamination, first by visual and tactile inspection and then a pH measurement (Hanna HI98128 pHep 5 Waterproof). Samples collected in this study had a pH range of 6.33 to 7.04, with these values being in accordance with expected pH values for contents extracted from the reticulo-rumen.

If the sample appeared to be contaminated, an additional sample was immediately collected. Subsequently, ruminal content samples were transferred into two 50-mL graduated containers to register the liquid and wet solid volume fractions collected and immediately placed in ice until storage at −80°C. Samples were later processed and analyzed for microbiome bacteria and archaea composition as described by Callahan et al. (2016). Sedative effects of the neuroleptic drug last approximately 15 min and the sampling procedure takes approximately 8 to 11 min in total (from neuroleptic administration up to sample collection). After the procedure was concluded, the manual pump and sampling tubing were carefully wiped with a paper towel and the tubing thoroughly washed and flushed with cold water 3 times, followed by an additional 2 flushes with distilled water. Once the cleaning process was concluded, the sampling tube was air-dried. A new sampling tube was used after 3 animals were sampled to minimize any potential risk of contamination in subsequent samples.

Individual DMI (Figure 2) and milk yield (**MY**; Figure 3) from the cows recruited in the study were recorded from −10 to +10 d relative to sampling. We did not observe detrimental effects of the intra-esophageal tube technique on DMI or MY between pre- and postsampling periods, with both continuing to increase over time as is the case for recently calved cows. Similarly, Klopp et al. (2018) did not report differences in DMI in dairy calves when an intra-esophageal tube technique was used for collection of rumen content samples.

**Figure 2:**
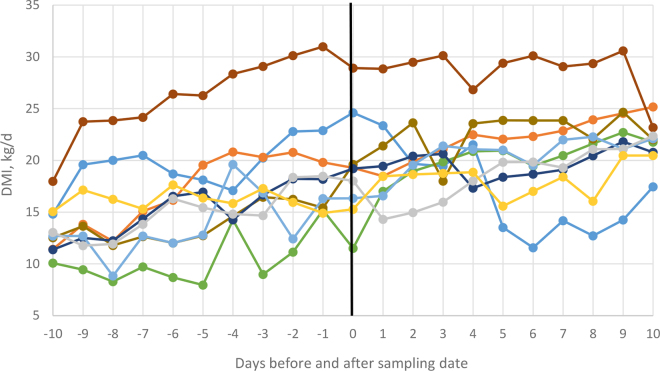
Individual cow DMI (kg/d); each line represents 1 of the 9 cows offered a diet with 46:54 forage-to-concentrate ratio sampled on d 0 (days before and after sampling) using the “Rumen Sampler MG.”

**Figure 3:**
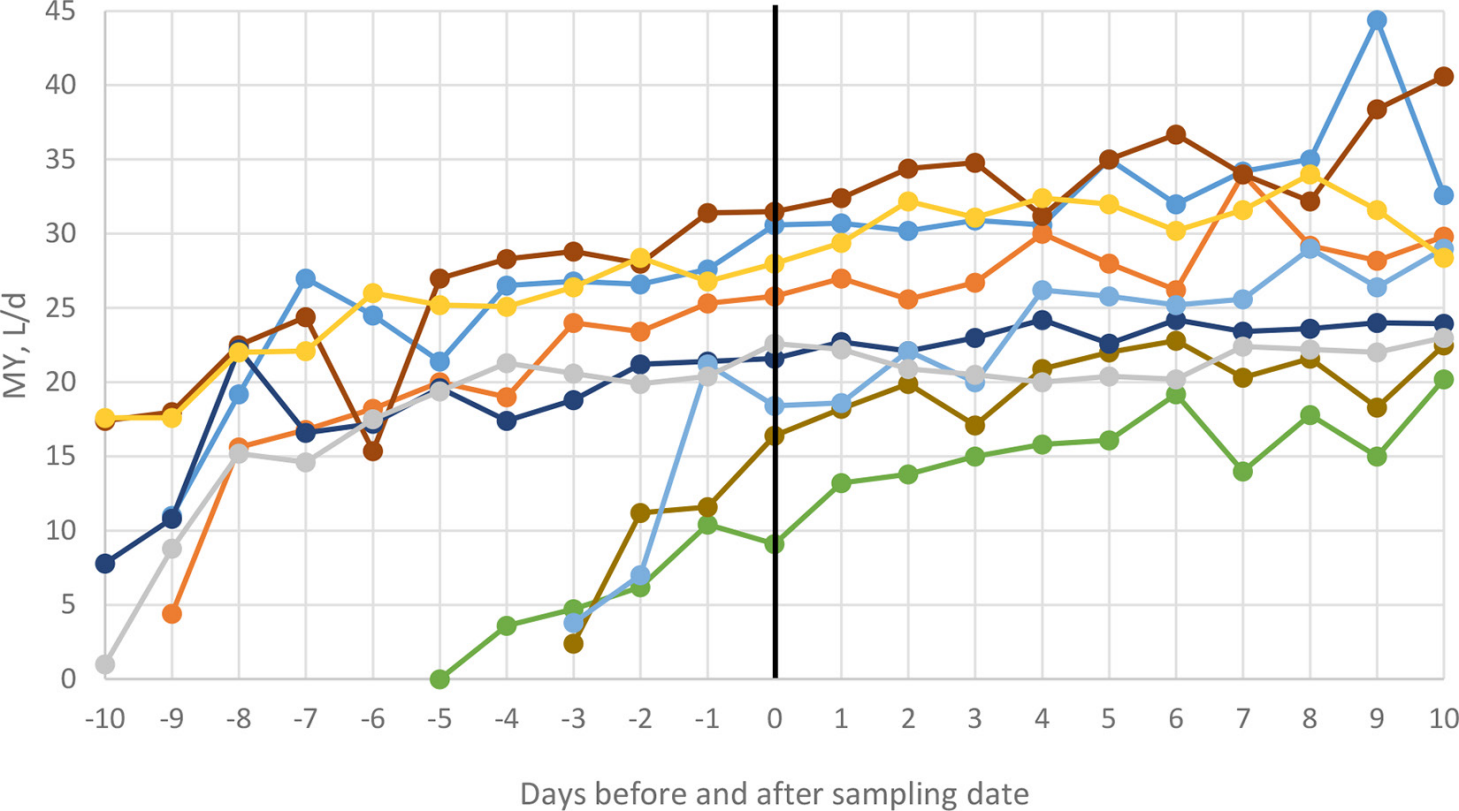
Individual cow milk yield (MY, L/d); each line represents 1 of the 9 cows offered a diet with 46:54 forage-to-concentrate ratio sampled on d 0 (days before and after sampling) using the “Rumen Sampler MG.”

One sample extraction from the Rumen Sampler MG can collect both liquid and solid rumen content fractions adequate for microbiome analysis (∼100 mL). At each extraction, the wet volume of particles recovered was approximately 35% to 40%. The wet volume of particles collected in our study represents a larger volume than that reported by Paz et al. (2016) who obtained 10% to 15% of wet volume of particles using an intra-esophageal tubing apparatus with a metal strainer for collection of ruminal content samples from lactating Holstein and Jersey dairy cows offered a diet with a forage-to-concentrate ratio of 51:49. In that study, the authors indicated that the technique used may over-represent the liquid fraction, and caution should be taken with this issue as the relative abundance of the rumen content–associated microbiome differs between fractions (de Menezes et al., 2011). For example, *Prevotella* genus is more abundant in the liquid fraction and its abundance was found to be increased in samples collected using previous intra-esophageal tubing techniques compared with samples collected via rumen fistula (Henderson et al., 2013). Ramos-Morales et al. (2014) reported differences in the abundance of *Fibrobacter succinogenes* between goat and sheep in samples collected using an intra-esophageal tubing technique, but no differences between those same animals in samples collected from rumen fistula. In our study, we accounted for 39 dominant taxa at genus level (relative abundance > 0.01%). At the phylum level, 41 taxa were identified and 28 were dominant, with *Firmicutes* (56.10%) and *Bacteroidetes* (23.63%) accounting for almost 80% of all taxa, followed by *Proteobacteria* (9.52%) and *Actinobacteria* (6.14%) (Miccoli et al., 2022). These findings are in accordance with those reported in the literature from samples collected from dairy cows via rumen fistula (Pitta et al., 2010; Jami and Mizrahi, 2012). The greater proportion of solids obtained with the Rumen Sampler MG technique compared with other intra-esophageal tubing techniques may allow for an improved characterization of the microbiome associated with the solid fraction and of the whole community in both liquid and solid fractions combined.

The device and technique described here represent an alternative to the traditional rumen cannulation technique and an improvement of intra-esophageal tubing techniques for collection of ruminal contents with greater recovery of solid fractions for improved characterization of the rumen microbiome community. The proposed sampling technique did not affect animal performance, suggesting that the technique imposed minimal stress on the animal.
